# Prenatal Vitamin D Supplementation and Child Respiratory Health: A Randomised Controlled Trial

**DOI:** 10.1371/journal.pone.0066627

**Published:** 2013-06-24

**Authors:** Stephen T. Goldring, Chris J. Griffiths, Adrian R. Martineau, Stephen Robinson, Christina Yu, Sheree Poulton, Jane C. Kirkby, Janet Stocks, Richard Hooper, Seif O. Shaheen, John O. Warner, Robert J. Boyle

**Affiliations:** 1 Department of Paediatrics, Imperial College London, London, United Kingdom; 2 Asthma UK Centre for Applied Research, Centre for Primary Care and Public Health, Barts and the London School of Medicine and Dentistry, Queen Mary University of London, London, United Kingdom; 3 Department of Medicine, Imperial College London, London, United Kingdom; 4 Portex Respiratory Unit, University College London, Institute of Child Health, London, United Kingdom; Research Center Borstel, Germany

## Abstract

**Background:**

Observational studies suggest high prenatal vitamin D intake may be associated with reduced childhood wheezing. We examined the effect of prenatal vitamin D on childhood wheezing in an interventional study.

**Methods:**

We randomised 180 pregnant women at 27 weeks gestation to either no vitamin D, 800 IU ergocalciferol daily until delivery or single oral bolus of 200,000 IU cholecalciferol, in an ethnically stratified, randomised controlled trial. Supplementation improved but did not optimise vitamin D status. Researchers blind to allocation assessed offspring at 3 years. Primary outcome was any history of wheeze assessed by validated questionnaire. Secondary outcomes included atopy, respiratory infection, impulse oscillometry and exhaled nitric oxide. Primary analyses used logistic and linear regression.

**Results:**

We evaluated 158 of 180 (88%) offspring at age 3 years for the primary outcome. Atopy was assessed by skin test for 95 children (53%), serum IgE for 86 (48%), exhaled nitric oxide for 62 (34%) and impulse oscillometry of acceptable quality for 51 (28%). We found no difference between supplemented and control groups in risk of wheeze [no vitamin D: 14/50 (28%); any vitamin D: 26/108 (24%) (risk ratio 0.86; 95% confidence interval 0.49, 1.50; P = 0.69)]. There was no significant difference in atopy, eczema risk, lung function or exhaled nitric oxide between supplemented groups and controls.

**Conclusion:**

Prenatal vitamin D supplementation in late pregnancy that had a modest effect on cord blood vitamin D level, was not associated with decreased wheezing in offspring at age three years.

**Trial Registration:**

Controlled-Trials.com ISRCTN68645785

## Introduction

Several observational studies have suggested a protective effect of higher maternal vitamin D intake during pregnancy on risk of wheezing [1]–[3], asthma and allergic rhinitis [4], and eczema [3] in offspring. Dietary intake of vitamin D makes a relatively small contribution to overall vitamin D status, and apparent effects of vitamin D intake may be confounded by other correlated nutrients. These studies did not report outcomes in relation to maternal serum 25-hydroxyvitamin D (25(OH)D) concentration, which takes account of skin synthesis, our primary source of vitamin D, and is the gold standard measure of vitamin D status [5], [6]. Observational studies of maternal or cord 25(OH)D levels and child health have yielded conflicting results, with higher 25(OH)D levels associated with increased risk of eczema and asthma [7], reduced risk of respiratory infection and preschool wheezing [8], and no association with asthma [8] or lung function [9] all reported.

There are plausible biological mechanisms for an association between prenatal 25(OH)D status and wheezing. Developmental programming of lung function and immune responses occurs during pregnancy [10], [11]. The active metabolite of vitamin D, 1,25-dihydroxyvitamin D, has been shown in animal and *in vitro* models to have potent immune effects [12] and a role in early lung development [13]. Current recommendations in the United Kingdom are for provision of 10 µg/day (400 IU/day) to all pregnant women [14]. Vitamin D insufficiency is nevertheless very common during pregnancy [15]–[17], and it has been proposed that prenatal vitamin D supplementation may prevent childhood wheezing and asthma [18]. We assessed children whose mothers had taken part in a randomised controlled trial of prenatal vitamin D supplementation, to determine whether supplementation prevented wheezing or allergic disease in the first 3 years of life [19].

## Methods

The protocol for this trial and supporting CONSORT checklist are available as supporting information; see Checklist S1 and Protocol S1.

### Ethics Statement

St. Mary’s Hospital Research Ethics Committee approved the follow up study (10/H0712/13) and women gave written informed consent to participation for themselves and their child. Where women only consented to interview over the telephone, verbal consent was taken in place of written consent and this was approved by the Ethics Committee. For each participant who gave verbal consent, this was taken and documented by 2 separate members of the research team.

### Study Subjects

This was a prospective follow up study of the offspring of women who took part in an ethnically stratified, parallel group, randomised controlled trial of vitamin D supplementation in pregnancy at St Mary’s Hospital London, a university-affiliated hospital prenatal clinic, between April and November 2007. The original trial was conducted to determine the effect of supplementation on 25(OH)D status of mothers in 4 specific ethnic groups, and their babies, at delivery [19]. Eligible participants were women presenting at 27 weeks gestation for routine glucose challenge test from the following ethnic groups: Asian, Middle Eastern, Black and White. Ethnic group was assigned based on participant self-report. Exclusion criteria were known sarcoidosis, osteomalacia, renal dysfunction or tuberculosis.

### Study Design

Women were randomised at 27 weeks gestation to no treatment (control), 800 IU ergocalciferol until delivery (daily), or a single oral dose of 200,000 IU cholecalciferol (bolus). The randomisation sequence was generated by an independent researcher using computer generated random number lists in blocks of 15, stratified by 4 ethnic groups in a 1∶1:1 ratio. The researcher gave participants a study number on entry to the trial, and treatment was allocated from the hospital pharmacy. Women were given instructions to swallow the tablets whole and to avoid other multivitamin supplements containing vitamin D. This trial was conducted before national guidance on routinely providing advice on vitamin D intake during pregnancy was introduced in March 2008 [14]. Investigators blind to original treatment allocation assessed offspring at three years of age using a validated health questionnaire and clinical assessment.

### Outcome Assessments

The primary outcome was prevalence of ‘wheeze ever’ as defined by the International Study of Asthma and Allergies in Childhood (ISAAC) [20]. Secondary outcomes were recurrent wheezing (≥2 episodes of reported wheezing since birth), wheeze in the year prior to assessment (ISAAC), wheeze with a positive asthma predictive index (loose criteria) [21], reported history of bronchodilator use, eczema ever (ISAAC), eczema in the year prior to assessment (ISAAC), allergic rhinitis, history of doctor diagnosed food allergy, history of lower respiratory tract infection (LRTI) (any of bronchiolitis, bronchitis, croup, pneumonia or an otherwise unspecified chest infection) and history of >4 episodes of upper respiratory tract infections (URTI) per year (defined as a positive answer to the question ‘how often does your child have an upper respiratory tract infection, with at least two of the following symptoms: cough, runny nose and fever?’).

Exhaled nitric oxide (eNO) was measured using an offline, tidal breathing technique adherent to ATS/ERS guidelines [22]. Impulse oscillometry (IOS) was performed in accordance with current international guidelines [23], before and 15 minutes after inhalation of 400 mcg salbutamol sulphate via spacer (Volumatic, Allen and Hanburys, Middlesex, UK). Resonant frequency, area under the reactance curve, and resistance at 10 and 20 Hz were chosen for data analysis. Bronchodilator responsiveness for each parameter was measured as the percentage change seen post-bronchodilator. See File S1 for description of IOS quality control and exhaled nitric oxide assessment.

Allergic sensitisation was defined as a skin prick test wheal at least 3 mm greater than the negative control (Glycerine) to one or more of the aeroallergens tested at 15 minutes, in the context of an appropriate response to the positive control (Histamine 10%). House dust mite, alternaria, cladosporium, cat, dog, grass pollen, silver birch pollen, peanut, milk and egg were tested (Stallergenes, Antony, France).

Serum total IgE (ImmunoCAP, Phadia, Uppsala, Sweden), 25(OH)D and eosinophil count were determined on the day of the child’s assessment. 25(OH)D was measured in mothers prior to randomisation, and in offspring at birth (cord blood) using a radio-immunoassay (DiaSorin, Stilwater, MN) in a clinical biochemistry laboratory that participates in the international Vitamin D external quality assessment program (DEQAS). Vitamin D deficiency was defined as 25(OH)D <25 nmol/L [24] and sufficiency ≥ 50 nmol/L [24], [25].

Primary health care records were obtained from participants’ general practitioners and reviewed by a single investigator (RJB), blinded to treatment allocation. Children were categorised as having ‘recurrent wheezing’ where ≥2 episodes of either wheezing, or respiratory distress treated with bronchodilator were recorded; ‘eczema’ if they had ≥2 attendances separated by ≥6 months where either topical corticosteroids were prescribed for treatment of an itchy skin rash, or a doctor’s diagnosis of eczema was made; and ‘food allergy’ if this diagnosis was recorded in any part of the primary healthcare record.

### Statistical Analysis

In the original study it was calculated that at least eight women in each ethnic group for each arm of treatment would be needed to demonstrate a significant difference in vitamin D levels at delivery in the no treatment group vs. the supplemented groups (power 90%, test of significance at 5% level). To account for drop-outs, preterm delivery, and delivery at another hospital, 15 women were allocated to each arm of treatment within each ethnic group (n = 180).

With 180 participants and 80% successful follow up, this study had 80% power with 2-sided alpha of 0.05 to detect a reduction in wheeze from 34% in the children of non-supplemented mothers [21] to 13% in the supplemented group. This level of risk reduction would be consistent with some observational studies [2], [21]. For primary analyses, bolus and daily vitamin D supplemented groups were analysed together and compared with the control group. We also report separate analyses of bolus and daily groups versus control, and of children born to mothers with vitamin D deficiency at enrolment (defined as 25(OH)D <25 nmol/L). Binary outcomes were analysed using risk ratios and odds ratios for unadjusted analysis, and logistic regression for adjusted analyses. Continuous outcomes were analysed using linear regression. Potential confounding factors used in the adjustment model were mother’s ethnic group, presence of household smokers, maternal smoking in pregnancy, exclusive breast-feeding to four months, any parental allergic history, any child vitamin supplementation, number of children in the household, age mother left full-time education and baseline concentration of 25(OH)D in maternal blood. For all analyses, participants were analysed in the treatment groups to which they were randomised.

Adjustment for multiple testing was performed using the Benjamini and Hochberg method [26]. This works by evaluating all the p-values from multiple analyses in order of significance, and determining whether each one can truly be counted as significant or not. For our analyses, there were 28 secondary clinical outcomes (14 for the daily group, and 14 for the bolus group) and the false discovery rate was controlled at 20%. All analyses were performed using SPSS version 19.0 (IBM, Chicago USA). Details of data handling and adjusted analysis are available in File S1.

## Results

**Figure 1:**
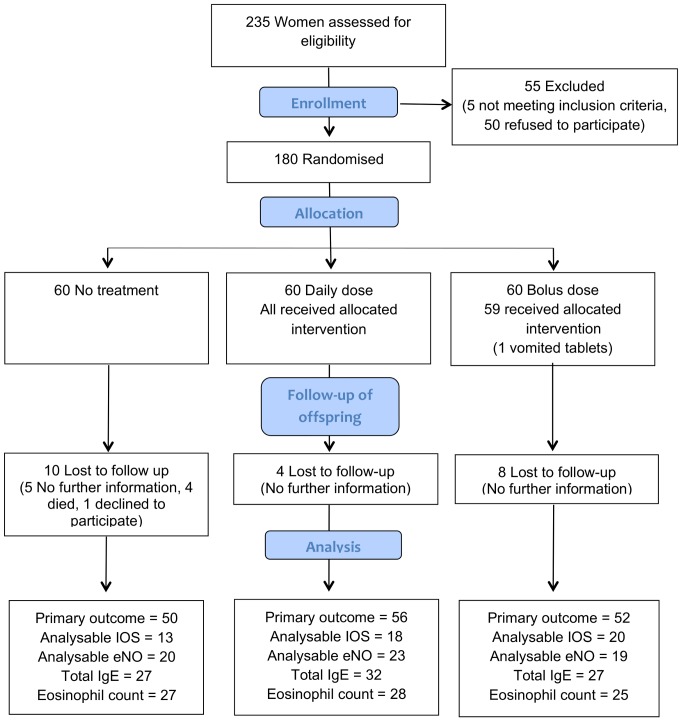
Recruitment and follow up of study participants.

Participant flow is shown in Figure 1. There was one twin pregnancy of whom only the first born was included in analysis. There were 4 deaths, all in the control group: one stillbirth at 41 weeks, one death aged two days (no further details available), one death aged 16 hours from meconium aspiration and one child with congenital abnormalities died post-operatively aged 17 months. We successfully assessed the primary outcome measure in 158 (88%) of the cohort at age 3 years, between July 2010 and February 2011. The follow up was stopped after all participants had been successfully contacted or were considered lost to follow up. 129 (72%) agreed to a review of their primary care record. Atopy was assessed by skin test for 95 children (53%), acceptable IOS data were available for 51 (28%), IgE level for 86 (48%), eNO level for 62 (34%) and eosinophil count for 80 (44%). Characteristics of the children and their mothers in each randomisation group were similar at baseline, as shown in Table 1. As previously reported [19], median cord 25(OH)D levels at delivery were significantly higher in supplemented children compared to the control group [control 17 nmol/l (interquartile range (IQR) 14–22); daily dose 26 nmol/l (IQR 17–45); *P*<0.001; bolus dose 25 nmol/l (IQR 18–34); *P*<0.001)].

**Table 1:** Characteristics of study participants followed up at age three years.

	Control	Daily vitamin D	Bolus vitamin D	Combined vitamin D
	(n = 50)	(n = 56)	(n = 52)	(n = 108)
Baseline maternal 25(OH)D <25 nmol/L, n/N (%)	24/50 (48)	25/56 (45)	22/52 (42)	47/108 (44)
Male sex, n/N (%)	27/50 (54)	32/56 (57)	26/52 (50)	58/108 (55)
Birth weight (g), mean (SD)	3268 (585)	3321 (525)	3290 (467)	3307 (497)
Ethnicity, n (%)				
Asian	12 (24)	15 (27)	13 (25)	28 (26)
Middle Eastern	13 (26)	14 (25)	14 (26)	28 (26)
Black	12 (24)	14 (25)	13 (25)	27 (25)
White	13 (26)	13 (23)	12 (24)	25 (23)
GA at delivery (weeks), mean (SD)	40 (1)	39 (2)	40 (1)	39 (2)
Nulliparous mother, n/N (%)	23/50 (46)	20/56 (36)	21/52 (40)	41/108 (38)
Vaginal delivery, n/N (%)	29/50 (58)	35/56 (63)	28/52 (54)	63/108 (58)
Maternal smoking during pregnancy, n/N (%)	2/48 (4)	3/55 (5)	0/46 (0)	3/101 (3)
Presence of household smokers, n/N (%)	16/48 (33)	15/55 (27)	17/46 (37)	32/101 (32)
Number of children in household, mean (SD)	2 (1)	2 (1)	2 (1)	2 (1)
Age mother left full time education, mean (SD)	20 (3)	20 (4)	21 (4)	21 (4)
Child in nursery, n/N (%)	27/48 (56)	36/55 (65)	20/45 (44)	57/101 (56)
Cat or dog in household, n/N (%)	4/48 (8)	5/54 (9)	4/46 (9)	9/100 (9)
Maternal Fitzpatrick skin score grade 3–6, n/N (%)	28/44 (64)	33/49 (67)	28/43 (65)	61/92 (66)
Maternal regular vitamin intake at three years, n/N (%)	9/46 (20)	16/53 (30)	16/45 (36)	32/98 (33)
At least one parent with allergic disease, n/N (%)	23/45 (51)	35/53 (66)	21/45 (47)	56/98 (57)
Exclusively breast-fed for 4 months, n/N (%)	22/48 (46)	20/54 (37)	23/44 (52)	43/98 (44)
Any child vitamin supplementation, n/N (%)	30/48 (63)	34/55 (62)	30/46 (65)	64/101 (63)
Completed immunizations to date, n/N (%)	48/49 (98)	54/55 (98)	43/47 (91)	97/102 (95)
Age at time of assessment (months), median (IQR)	37.9 (36.9, 39.9)	37.1 (36.5, 38.8)	37.4 (36.5, 39.5)	37.3 (36.5, 39.0)
Child Fitzpatrick skin score grade 3–6, n (%)	29/42 (69)	34/48 (71)	29/43 (67)	63/91 (69)
Child BMI Z score age 3, mean (SD)	0.51 (1.48)	0.35 (1.15)	0.62 (1.15)	0.47 (1.15)
Child outdoors >1 hour/day, n/N (%)	32/48 (67)	34/53 (64)	33/45 (73)	67/98 (68)
Child TV/computer >2 hours/day, n/N (%)	24/48 (50)	18/53 (34)	17/45 (38)	35/98 (36)
Child 25(OH)D (nmol/L) age 3, median (IQR)	42 (27, 68)	35 (21, 66)	42 (30, 93)	41 (26, 72)

Data shown are for all participants analysed.

IQR = interquartile range, SD = standard deviation, GA = gestational age, SCBU = Special Care Baby Unit, BMI = Body mass index.

### Effect of Prenatal Vitamin D Supplementation on Parent Reported Wheezing, Allergic Disease and Respiratory Infections

We found no significant difference in the primary outcome of ‘wheeze ever’ between treatment groups (any vitamin D vs. control Table 2, daily vs. control Table 3, bolus vs. control Table 4). There was also no difference between groups in prevalence of eczema or atopy. We found significantly increased bronchodilator use and higher prevalence of LRTI’s in the offspring of the bolus group, but this did not remain significant after adjusting for multiple testing at a 20% threshold. File S1 shows complete analyses of these outcomes for the offspring of mothers with vitamin D deficiency at baseline (any vitamin D vs. control, Table S1 in File S1, daily vs. control, Table S2 in File S1, bolus vs. control, Table S3 in File S1). We found no significant difference in the primary outcome of ‘wheeze ever’, or for any secondary outcomes between these groups.

**Table 2:** Clinical outcomes at age three years. Combined vitamin D groups versus control.

	Control	Combined vitamin D	RR	OR	*P*	aOR†	*P*
	(%)	(%)	(95% CI)	(95% CI)		(95% CI)	
Wheeze ever	14/50 (28)	26/108 (24)	0.86 (0.49, 1.50)	0.82 (0.38, 1.74)	0.60	0.73 (0.33, 1.65)	0.45
Recurrent wheezing	7/50 (14)	17/108 (16)	1.12 (0.50, 2.54)	1.15 (0.44, 2.97)	0.77	1.14 (0.42, 3.13)	0.80
Wheezing in the last year	8/50 (16)	17/108 (16)	0.98 (0.46, 2.13)	0.98 (0.39, 2.45)	0.97	0.95 (0.36, 2.51)	0.92
Wheeze with positive API	7/50 (15)	15/107 (14)	1.00 (0.44, 2.30)	1.00 (0.38, 2.64)	1.00	1.06 (0.37, 3.02)	0.91
Any bronchodilator use	4/49 (8)	24/104 (23)	2.83 (1.04, 7.71)	3.37 (1.10, 10.34)	0.03*	3.14 (0.96, 10.28)	0.06*
Eczema ever	15/49 (31)	30/102 (29)	0.96 (0.57, 1.61)	0.75 (0.35, 1.62)	0.47	0.72 (0.32, 1.61)	0.42
Eczema in the last year	7/49 (14)	20/103 (19)	1.36 (0.62, 3.00)	1.34 (0.52, 3.47)	0.55	1.52 (0.55, 4.21)	0.42
Atopy	7/27(26)	11/68 (16)	0.62 (0.27, 1.44)	0.56 (0.19, 1.66)	0.30	0.29 (0.07, 1.16)	0.08
Allergic rhinitis	7/49 (14)	11/101 (12)	0.76 (0.31, 1.85)	0.63 (0.22, 1.78)	0.38	0.69 (0.22, 2.13)	0.52
Food allergy diagnosis	3/49 (6)	12/102 (12)	1.92 (0.57, 6.50)	1.81 (0.48, 6.85)	0.38	1.93 (0.45, 8.29)	0.38
>4 URTI/year	7/50 (14)	20/103 (19)	1.39 (0.63, 3.06)	1.43 (0.55, 3.68)	0.46	1.34 (0.49, 3.68)	0.57
LRTI ever	11/50 (22)	31/101 (31)	1.40 (0.77, 2.54)	1.47 (0.64, 3.37)	0.37	1.60 (0.67, 3.85)	0.29
Primary health care records							
Recurrent wheeze	3/31 (10)	10/70 (14)	1.48 (0.44, 5.00)	1.56 (0.40, 6.10)	0.52	1.47 (0.33, 6.55)	0.61
Eczema	6/31 (19)	10/70 (14)	0.74 (0.29, 1.85)	0.46 (0.23, 2.12)	0.52	0.63 (0.18, 2.16)	0.46
Food allergy	2/31 (6)	2/70 (3)	0.44 (0.07, 3.00)	0.43 (0.06, 3.18)	0.58	0.14 (0.00, 4.39)	0.26

RR = risk ratio, OR = unadjusted odds ratio, aOR =  adjusted odds ratio, API = Asthma predictive index, URTI = upper respiratory tract infection, LRTI = lower respiratory tract infection.

† Model adjusted for mother’s ethnic group, presence of household smokers, maternal smoking in pregnancy, exclusive breast-feeding to four months, any parental allergic history, any child vitamin supplementation, number of children in the household, age mother left full-time education and baseline concentration of 25(OH)D in maternal blood. n = 139 for primary outcome measure.

* Adjusted p-value for multiple testing non-significant at 20% level.

**Table 3:** Clinical outcomes at age three years. Daily vitamin D versus control.

	Control	Daily Vitamin D	RR	OR	*P*	aOR†	*P*
	(%)	(%)	(95% CI)	(95% CI)		(95% CI)	
Wheeze ever	14/50 (28)	11/56 (20)	0.70 (0.35, 1.40)	0.63 (0.26, 1.55)	0.31	0.56 (0.20, 1.57)	0.27
Recurrent wheezing	7/50 (14)	8/56 (14)	1.02 (0.40, 2.61)	1.02 (0.34, 3.06)	0.97	1.11 (0.32, 3.92)	0.87
Wheezing in the last year	8/50 (16)	8/56 (14)	0.89 (0.36, 2.20)	0.88 (0.30, 2.54)	0.81	0.88 (0.27, 2.90)	0.84
Wheeze with positive API	7/50 (14)	6/56 (11)	0.77 (0.28, 2.13)	0.74 (0.23, 2.36)	0.61	0.65 (0.15, 2.82)	0.57
Any bronchodilator use	4/49 (8)	10/56 (18)	2.23 (0.75, 6.67)	2.45 (0.72, 8.37)	0.15	2.24 (0.54, 9.33)	0.27
Eczema ever	15/49 (31)	15/54 (28)	0.91 (0.50, 1.66)	0.87 (0.37, 2.04)	0.75	0.61 (0.23, 1.67)	0.34
Eczema in the last year	7/49 (14)	11/55 (20)	1.40 (0.59, 3.33)	1.50 (0.53, 4.23)	0.44	1.31 (0.38, 4.54)	0.67
Atopy	7/27 (26)	4/36 (11)	0.41 (0.13, 1.27)	0.36 (0.09, 1.38)	0.13	0.36 (0.05, 2.94)	0.34
Allergic rhinitis	7/49 (14)	7/55 (13)	0.89 (0.34, 2.36)	0.88 (0.28, 2.70)	0.82	0.63 (0.17, 2.36)	0.49
Food allergy diagnosis	3/49 (6)	8/55 (15)	2.38 (0.67, 8.46)	2.61 (0.65, 10.45)	0.16	4.53 (0.52, 39.33)	0.17
>4 URTI/year	7/50 (14)	11/55 (20)	1.43 (0.60, 3.40)	1.54 (0.55, 4.33)	0.42	1.39 (0.41, 4.68)	0.60
LRTI ever	11/50 (22)	14/54 (26)	1.18 (0.59, 2.35)	1.24 (0.50, 3.07)	0.64	1.00 (0.35, 2.91)	1.00
Primary health care records							
Recurrent wheeze	3/31 (10)	4/36 (11)	1.15 (0.28, 4.74)	1.17 (0.24, 5.67)	0.85	1.43 (0.21, 9.89)	0.71
Eczema	6/31 (19)	5/36 (14)	0.72 (0.24, 2.12)	0.67 (0.18, 2.46)	0.55	0.61 (0.13, 2.86)	0.53
Food allergy	2/31 (7)	2/36 (6)	0.86 (0.13, 5.76)	0.85 (0.11, 6.44)	0.88	**-**	**-**

RR = Risk ratio, OR = unadjusted odds ratio, aOR =  adjusted odds ratio, API = Asthma predictive index, URTI = upper respiratory tract infection, LRTI = lower respiratory tract infection.

† Model adjusted for mother’s ethnic group, presence of household smokers, maternal smoking in pregnancy, exclusive breast-feeding to four months, any parental allergic history, any child vitamin supplementation, number of children in the household, age mother left full-time education and baseline concentration of 25(OH)D in maternal blood.

**Table 4:** Clinical outcomes at age three years.

	Control	Bolus Vitamin D	RR	OR	*P*	aOR†	*P*
	(%)	(%)	(95% CI)	(95% CI)		(95% CI)	
Wheeze ever	14/50 (28)	15/52 (29)	1.03 (0.56, 1.91)	1.04 (0.44, 2.47)	0.93	1.17 (0.44, 3.10)	0.75
Recurrent wheezing	7/50 (14)	9/52 (17)	1.24 (0.50 (3.07)	1.29 (0.44, 3.77)	0.65	1.91 (0.53, 6.75)	0.31
Wheezing in the last year	8/50 (16)	9/52 (17)	1.08 (0.45, 2.58)	1.10 (0.39, 3.12)	0.86	1.29 (0.40, 4.15)	0.66
Wheeze with positive API	7/50 (14)	9/51 (18)	1.26 (0.51, 3.12)	1.32 (0.45, 3.86)	0.62	2.01 (0.58, 6.99)	0.27
Any bronchodilator use	4/49 (8)	14/48 (29)	3.57 (1.27, 10.09)	4.63 (1.40, 15.34)	0.008*	5.43 (1.39, 21.20)	0.02*
Eczema ever	15/49 (31)	15/48 (31)	1.02 (0.56, 1.85)	1.03 (0.44, 2.44)	0.95	0.86 (0.32, 2.28)	0.76
Eczema in the last year	7/49 (14)	9/48 (19)	1.31 (0.53, 3.24)	1.39 (0.47, 4.08)	0.55	1.97 (0.47, 8.27)	0.36
Atopy	7/27 (26)	7/32 (22)	0.84 (0.34, 2.11)	0.80 (0.24, 2.66)	0.72	0.31 (0.05, 1.83)	0.20
Allergic rhinitis	7/49 (14)	4/46 (9)	0.61 (0.19, 1.94)	0.57 (0.16, 2.10)	0.40	0.91 (0.20, 4.11)	0.90
Food allergy diagnosed	3/49 (6)	4/47 (9)	1.36 (0.32, 5.78)	1.43 (0.30, 6.75)	0.65	1.31 (0.24, 7.18)	0.76
>4 URTI/year	7/50 (14)	9/48 (19)	1.34 (0.54, 3.31)	1.42 (0.48, 4.17)	0.53	1.72 (0.49, 6.04)	0.39
LRTI ever	11/50 (22)	17/47 (36)	1.64 (0.86, 3.14)	2.01 (0.82, 4.92)	0.12	2.87 (1.03, 8.03)	0.05*
Primary health care record							
Recurrent wheeze	3/31 (10)	6/34 (18)	1.82 (0.50, 6.68)	2.00 (0.46, 8.80)	0.35	1.85 (0.36, 9.66)	0.47
Eczema	6/31 (19)	5/34 (15)	0.76 (0.26, 2.24)	0.72 (0.20, 2.64)	0.62	0.63 (0.11, 3.59)	0.60
Food allergy	2/31 (7)	0/34 (0)	0.0 (-, -)	**-**	**-**	**-**	**-**

Bolus vitamin D versus control.

RR = Risk Ratio, OR = unadjusted odds ratio, aOR =  adjusted odds ratio, API = Asthma predictive index, URTI = upper respiratory tract infection, LRTI = lower respiratory tract infection.

† Model adjusted for mother’s ethnic group, presence of household smokers, maternal smoking in pregnancy, exclusive breast-feeding to four months, any parental allergic history, any child vitamin supplementation, number of children in the household, age mother left full-time education and baseline concentration of 25(OH)D in maternal blood.

* Adjusted p-value for multiple testing non-significant at 20% level.

Since 22 offspring were not followed up, sensitivity analyses were performed to determine if this influenced the result for the primary outcome. Two scenarios were considered – the first where no drop-outs wheezed and the second where all drop-outs wheezed (Table S4 in File S1). There was no significant difference between groups in wheezing prevalence for either of these scenarios.

For 122 children assessed at 3 years, cord blood 25(OH)D levels were available. We found no difference between the natural logarithm (Ln) of cord blood 25(OH)D levels in children with and without a history of wheeze, atopy, eczema, any LRTI or >4 episodes of URTI per year (Table S5 in File S1). There was also no correlation between Ln cord blood 25(OH)D level and Ln Total IgE at age three years for 68 children (Beta coefficient = −0.05, P = 0.72).

### Effect of Prenatal Vitamin D Supplementation on Lung Function and Bronchodilator Responsiveness

**Figure 2:**
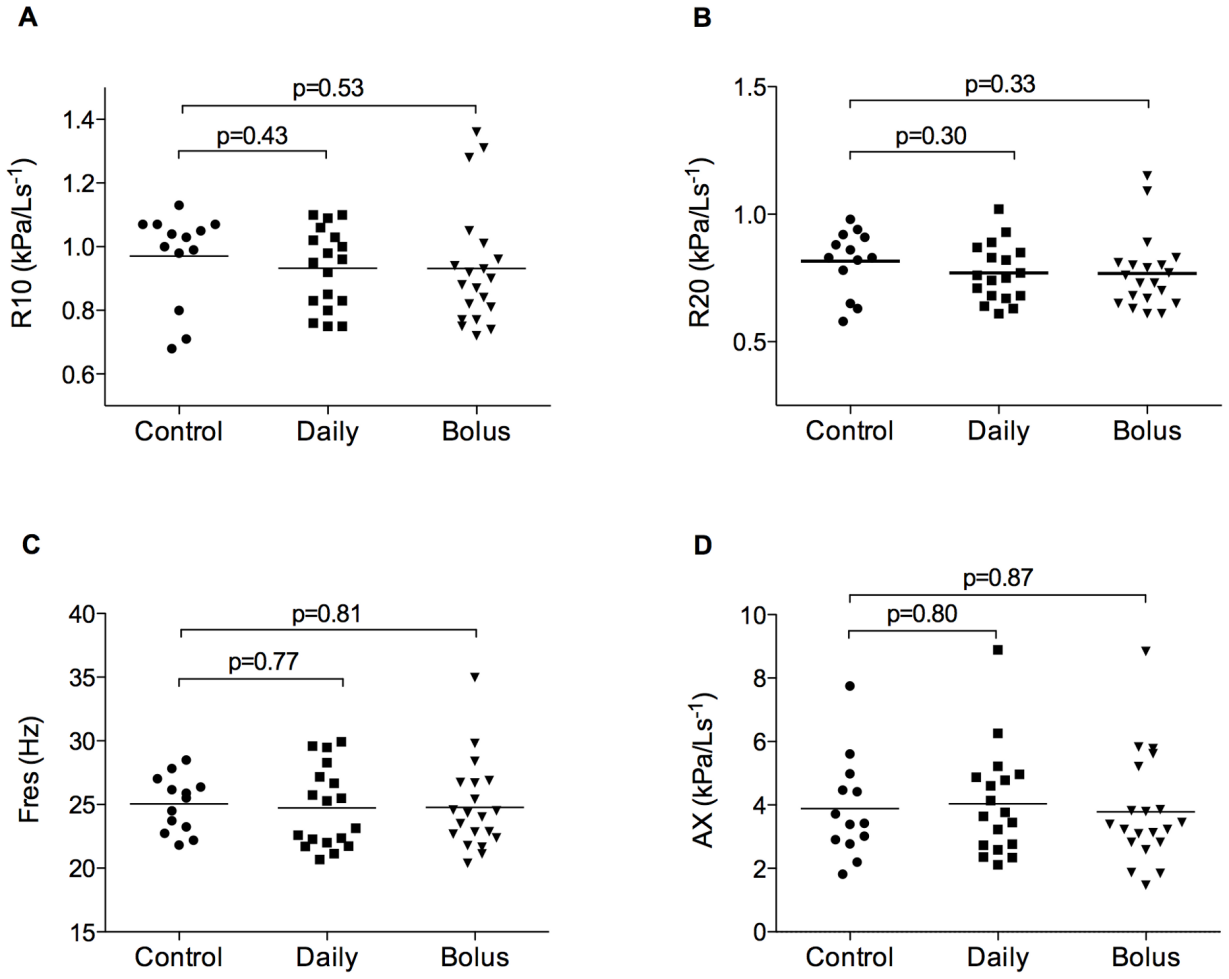
Baseline respiratory resistance at 10 Hz (R10, A), 20 Hz (R20, B), resonant frequency (F_res_, C) and area under the reactance curve (AX, D) for control (n = 13), daily (n = 18), and bolus (n = 20) vitamin D groups at age three years. Horizontal bars represent means. No significant difference between groups.

**Figure 3:**
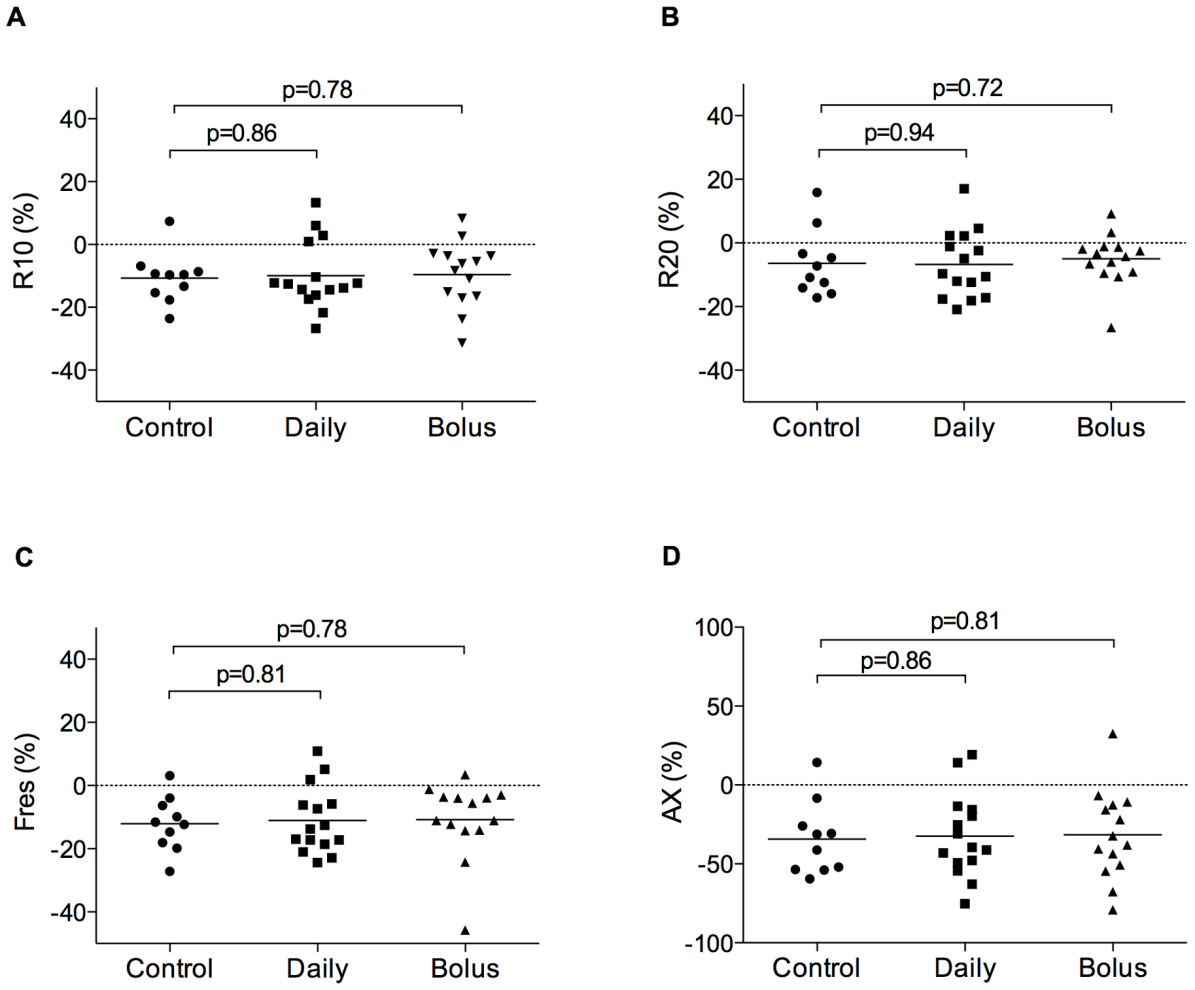
Bronchodilator response for respiratory resistance at 10 Hz (R10, A), 20 Hz (R20, B), resonant frequency (F_res_, C) and area under the reactance curve (AX, D) for control (n = 10), daily (n = 15), and bolus (n = 14) vitamin D groups at age three years. Horizontal bars represent means. No significant difference between groups.

Fifty-one of 180 (28%) children provided acceptable IOS data for analyses. We found no significant difference between groups in baseline respiratory resistance at 10 or 20 Hz, resonant frequency, area under the reactance curve or percentage response of these parameters to bronchodilator, when data were analysed for the two forms of prenatal vitamin D supplementation combined (Table S6 in File S1), or separately (Figures 2 and 3).

### Effect of Prenatal Vitamin D Supplementation on Objective Measures of Allergic Inflammation

**Figure 4:**
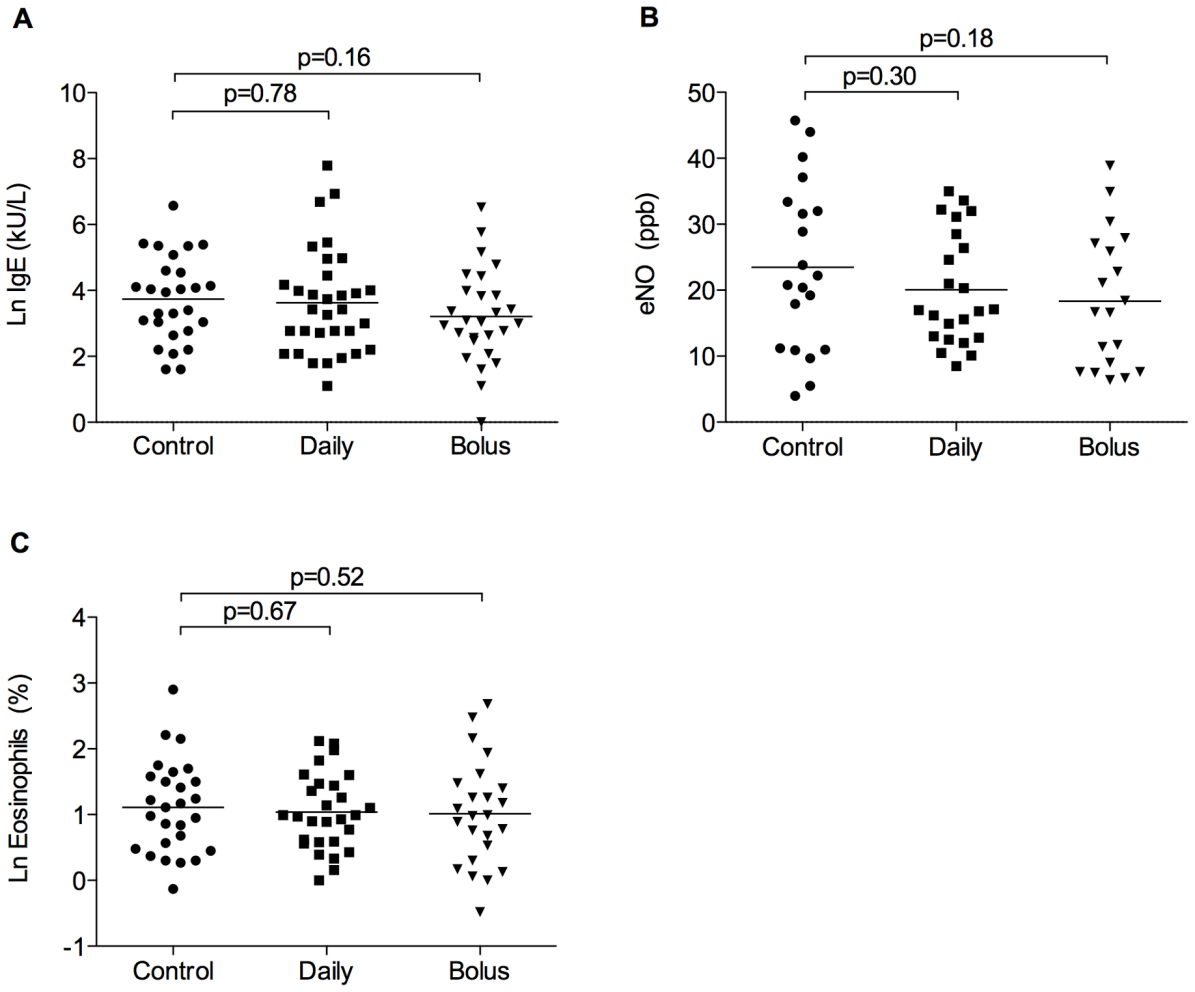
Measures of allergic inflammation at age three years. Ln IgE (A; n = 86), eNO (B; n = 62), and Ln eosinophil count (C; n = 90). Control versus daily and bolus vitamin D. Horizontal bars represent means. No significant difference between groups.

Total IgE levels, eNO and eosinophil counts were available for 106/180 (59%), 62/180 (34%) and 80/180 (44%) of offspring. We found no significant difference between groups in these outcomes assessed at age 3, when analysed for the two forms of prenatal vitamin D supplementation combined (Table S7 in File S1) or analysed separately (Figure 4).

### Adverse Events

No adverse events related to treatment were reported in the offspring.

## Discussion

In this randomised controlled trial of vitamin D supplementation in late pregnancy in a relatively deficient mixed race population, daily supplementation with 800 IU ergocalciferol or a single bolus dose of 200,000 IU cholecalciferol from 27 weeks gestation did not prevent wheezing, allergic disease or influence measures of lung function or allergic inflammation in offspring in the first three years of life. We were unable to confidently exclude an effect on some secondary outcome measures such as atopy at 3 years, where statistical power was limited. Our results suggest that the most likely explanation for the finding of decreased wheezing risk in offspring of mothers with a higher vitamin D intake during pregnancy in observational studies [1]–[4], is confounding by other dietary or lifestyle factors associated with vitamin D intake.

Our study has a number of limitations. First, we relied on a subjective primary outcome measure and participants were not blind to treatment allocation, which introduces a risk of reporter bias. Investigators were, however, kept blind to treatment allocation until database lock. Moreover the results of subjective primary outcome measures were supported by the results of both our secondary objective outcome assessments and a blinded assessment of prospectively recorded healthcare records. Second, this was a small study with limited statistical power, particularly for detecting differences in objective outcomes. Our power calculation was based on the study by Camargo et al [2], which estimated a risk reduction for recurrent wheezing from 34% to 13% comparing mothers with the highest quartile of daily vitamin D intake (median 724 IU) to those in the lowest quartile of intake (median 356 IU). However the prevalence of wheeze in our control group was 28% thus reducing the power of the study. The 95% confidence interval for our primary outcome measure was 0.49 to 1.50, so we are unable to confidently exclude effects of prenatal vitamin D supplementation on risk of wheezing, or on the development of atopy especially in mothers treated with daily vitamin D. Third, we did not assess vitamin D receptor polymorphisms or other potentially important genotypic variations. It is possible that vitamin D supplementation is effective for preventing wheezing or atopy in the presence of specific maternal or infant genotypes, as suggested by others [27]. Fourth, women from four ethnic groups were enrolled in this study, with significant differences in baseline vitamin D levels between Caucasian women and all other ethnic groups (data not shown). Previous observational studies have predominantly studied Caucasians [2]. Although randomisation in this trial was stratified by ethnicity, and we included baseline vitamin D in our adjusted model, it is possible that this source of heterogeneity could have masked positive findings. Fifth, supplementation with vitamin D only started at 27 weeks of gestation. It is known that immune cells develop much earlier in fetal life [28], and that airway development to the respiratory bronchioles is complete by 16 weeks gestation, [29]. It may be that supplementation earlier in pregnancy, or indeed pre-conception, is necessary for protection against childhood wheezing.

Finally, we are also unable to exclude the possibility that vitamin D supplementation at a higher dose, might protect against early childhood wheezing. Although the vitamin D doses used in this trial were greater than the current recommended intake for pregnant women of 400 IU/day during pregnancy in the United Kingdom [14], [17], and 600 IU in the United States [25], only a small percentage of offspring had cord 25(OH)D levels in the sufficient range (13% daily group, 3% bolus group). It has recently been shown that doses of 4000 IU of vitamin D daily are safe and effective for short term treatment of vitamin D deficiency during pregnancy [30], although the long term effects on child health have not been studied. Two on-going trials, the Vitamin D Prenatal Asthma Reduction Trial (NCT00920621), and ABCvitaminD (NCT00856947) are specifically exploring the effects of earlier and higher dose prenatal vitamin D supplementation on child health.

Two recent observational studies that documented prenatal vitamin D status by measuring cord blood 25(OH)D concentration, found relationships with allergic sensitisation and/or wheezing in early childhood [8], [31]. When we analysed our own data on cord blood 25(OH)D concentration and wheezing, atopy, total IgE level and frequency of respiratory infection in the first three years, no significant relationship was found. While our interventions did result in at least 50% higher cord blood 25(OH)D concentrations compared with no treatment, cord blood levels were still significantly lower in the intervention groups than in these studies. The results of our randomised controlled trial do not support a strong causal relationship between low prenatal vitamin D status within the deficient/insufficient range and increased risk of allergic sensitization or wheezing in early childhood. We are unable to confidently exclude a similar relationship in more vitamin D sufficient populations [8].

The findings of our study are specific to a population of vitamin D deficient women (half had baseline 25(OH)D levels below 25 nmol/L) and two specific forms of prenatal vitamin D supplementation. The doses of supplementation were chosen pragmatically, were not stratified based on individual vitamin D status or genotype, and response to treatment was not monitored before delivery. The study population was not selected for allergy or asthma risk and, as a mixed race urban population, is representative of many populations of pregnant women worldwide [32]. Although these findings cannot automatically be generalised to other populations of pregnant women, one might expect prenatal vitamin D supplementation to show its clearest clinical effects in a deficient population such as this.

In summary we found no evidence for a protective effect of prenatal vitamin D supplementation from 27 weeks gestation on childhood wheezing, allergic disease, lung function or markers of airway inflammation in the first 3 years of life. Given the modest effects on cord blood vitamin D achieved by these interventions, the safety and efficacy of higher dose prenatal vitamin D supplementation strategies need to be explored.

## Supporting Information

Checklist S1 **CONSORT Checklist.**
(DOC)

File S1 **Supporting Information.**
(DOC)

Protocol S1 **Trial Protocol.**
(DOCX)
