# Supplementary material for: Prenatal Vitamin D Supplementation and Child Respiratory Health: A Randomised Controlled Trial
Source: PLoS One. 2013 Jun 24;8(6):e66627. doi: 10.1371/journal.pone.0066627 (PMC3691177; doi:10.1371/journal.pone.0066627)
Supplement: File S1 — Supporting Information. (DOC) [file pone.0066627.s002.doc]

**Prenatal vitamin D supplementation and child respiratory health: a randomised controlled trial**

^1^Stephen T Goldring, ^2^Chris J Griffiths, ^2^Adrian R Martineau, ^1^Stephen Robinson, ^1^Christina Yu, ^1^Sheree Poulton, ^3^Jane C Kirkby, ^3^Janet Stocks, ^2^Richard Hooper, ^2^Seif O Shaheen, ^1^John O Warner, ^1^Robert J Boyle

^1^Department of Paediatrics, Imperial College London, United Kingdom

^2^Asthma UK Centre for Applied Research, Centre for Primary Care and Public Health, Barts and the London School of Medicine and Dentistry, Queen Mary University of London, United Kingdom

^3^Department of Medicine, Imperial College London, United Kingdom

^4^Portex Respiratory Unit, University College London, Institute of Child Health, London, United Kingdom

Corresponding author:

Robert J Boyle

Senior Lecturer in Paediatrics

Imperial College London

Wright Fleming Building

Norfolk Place

London W2 1PG

United Kingdom

Tel: +44 207 594 3990

Fax: +44 207 594 3984

Email: r.boyle@nhs.net

**Supporting Information**

**Methods**

**Impulse oscillometry (IOS) quality control**

Impulse oscillometry (IOS) was performed in accordance with ERS/ATS task force guidelines , using the Jaeger IOS system (Wurzburg, Germany), before and 15 minutes after inhalation of 400mcg salbutamol sulphate via a large volume spacer (Volumatic, Allen and Hanbury, Middlesex, UK). In addition, the following online quality assurance protocol was adopted (personal communication from the late Michael Goldman): Support of the cheeks to ensure no movement of the mouth (chewing, talking etc.) and thereby minimise upper-airway compliance; observation of the tidal volume time-based trace to ensure it was stable, free from drift with no hyper- or hypo- ventilation; observation of the impedance at 5Hz trace to ensure consistency throughout the measurement; ensuring no obvious spikes in impedance due to swallowing, glottic closure or cough were present. If these criteria were not met, the measurement was stopped and the technique reviewed.

On completion of all data collection for the study, two trained operators (STG, JCK) assessed all readings. Essential acceptance criteria for a reading to be included in analyses were as follows: at least 10 seconds in duration with a minimum of four tidal breaths; stable, regular tidal breaths, free from drift; no obvious abnormalities, recording appears ‘physiologically plausible’ (i.e. physiological implausible data identified and excluded); coherence >0.4 at 5Hz and >0.7 at 10 Hz. Results were rejected if there was evidence of tongue position artefact, (noted by a parallel increase in resistance at all frequencies).

For each child, the reported result was the mean of 3 to 5 “acceptable” (as defined above) measurements .

**Exhaled nitric oxide equipment**

Exhaled nitric oxide was measured using an offline, tidal breathing technique in accordance with the principles recommended by the ATS/ERS as follows: measurements were made before other lung function tests and before administration of salbutamol; subjects breathed through a two-way non-rebreathing mask with partitioned nose and mouth chambers (Series 7975, PED SM M/F Mask Yshp, Han Rudolph, Kansas City, USA); subjects breathed normally from a source of nitric oxide free air (<5ppb) (NO scrubber, Sievers reorder P/N: AFL 01410-01, NORTH part no. N7500-2, USA, 40700842 rev E) and exhaled against 5cm H_2_0 resistance (Model 7100R, flow range 0 to 2 L/sec, SN 710-1227, Han Rudolph, Kansas City, USA) to prevent contamination of the sample with nasal NO; the initial 10 exhalations were discarded to allow for washout of dead space of the lungs; 5 exhalations were collected into a NO-inert bag (Maximum pressure 12 cmH20, Series 6000, P/N CR1735, 10x10 outside measurement, 1L capacity, Hans Rudolph, Kansas City, USA) connected via a three way valve (2100 series 3 way stopcock, Han Rudolph, Kansas City, USA); additional samples were collected after 30 seconds of tidal breathing in ambient air; 3 samples were collected ideally, with the median value of at least two measurements was used for data analysis; samples were analysed within 1 hour of collection using the NIOX flex (Aerocrine, Sweden) with the following settings - 7 seconds collection time, flow rate of 50mls/s , calculation segment 50% to 100% of measurement.

**Statistical methods**

All data were double entered onto a database and underwent range checks, data cleaning and checking with source documents before database lock. A statistical analysis plan was agreed between all the investigators prior to unblinding. Where necessary data were natural log transformed for analysis. All analyses presented here were specified in the statistical analysis plan, and no post-hoc analyses were undertaken.

For adjusted analyses, treatment effects were adjusted for variables thought to influence risk of wheezing or postnatal vitamin D status. These were mother’s ethnic group, presence of household smokers, maternal smoking in pregnancy, exclusive breast-feeding to four months, any parental allergic history, any child vitamin supplementation, number of children in the household, age mother left full-time education, baseline concentration of 25(OH)D in maternal blood. Analyses were based on all participants with complete data.

**Tables**

Table S1. Clinical outcomes at age three years in offspring of mothers with baseline 25(OH)D <25nmol/L.

**Combined vitamin D versus control.**

|  | Control | Combined vitamin D | RR | OR | *P* | aOR^†^ | *P* |
| --- | --- | --- | --- | --- | --- | --- | --- |
|  | (%) | (%) | (95% CI) | (95% CI) |  | (95% CI) |  |
| Wheeze ever | 7/24 (29) | 11/47 (23) | 0.80 (0.36, 1.80) | 0.74 (0.25, 2.25) | 0.60 | 0.55 (0.15, 2.05) | 0.38 |
| Recurrent wheezing | 4/24 (17) | 8/47 (17) | 1.02 (0.34, 3.05) | 1.03 (0.28, 3.82) | 0.97 | 0.97 (0.23, 4.02) | 0.97 |
| Wheezing in the last year | 5/24 (21) | 8/47 (17) | 0.82 (0.30, 2.23) | 0.78 (0.23, 2.71) | 0.69 | 0.68 (0.17, 2.67) | 0.58 |
| Wheeze with positive API | 4/24 (17) | 6/46 (13) | 0.78 (0.24, 2.51) | 0.75 (0.19, 2.96) | 0.68 | 0.68 (0.15, 3.07) | 0.62 |
| Any bronchodilator use | 2/24 (8) | 9/44 (21) | 2.46 (0.58, 10.46) | 2.83 (0.56, 14.33) | 0.20 | 2.76 (0.37, 20.54) | 0.32 |
| Eczema ever | 8/24 (33) | 14/43 (33) | 0.98 (0.48, 1.99) | 0.97 (0.33, 2.79) | 0.95 | 0.51 (0.15, 1.80) | 0.30 |
| Eczema in the last year | 4/24 (17) | 10/44 (23) | 1.36 (0.48, 3.89) | 1.47 (0.41, 5.31) | 0.56 | 1.09 (0.24, 4.99) | 0.91 |
| Atopy | 4/13 (31) | 4/27 (15) | 0.48 (0.14, 1.63) | 0.39 (0.08, 1.91) | 0.24 | **-** | **-** |
| Allergic rhinitis | 3/24 (13) | 4/44 (9) | 0.73 (0.18, 2.99) | 0.70 (0.14, 3.42) | 0.66 | 0.44 (0.06, 3.47) | 0.43 |
| Food allergy diagnosis | 2/24 (8.3) | 5/44 (11.4) | 1.36 (0.29, 6.51) | 1.41 (0.25, 7.88) | 0.69 | 0.06 (0.001, 3.80) | 0.18 |
| > 4 URTI/year | 3/24 (13) | 12/44 (27) | 2.18 (0.68, 6.99) | 2.63 (0.66, 10.43) | 0.16 | 2.15 (0.45, 10.32) | 0.34 |
| LRTI ever | 7/24 (29) | 13/43 (30) | 1.04 (0.48, 2.24) | 1.05 (0.35, 3.15) | 0.93 | 0.97 (0.27, 3.51) | 0.96 |
| Primary health care record |  |  |  |  |  |  |  |
| Recurrent wheeze | 1/17 (6) | 5/29 (17) | 2.93 (0.37, 23.05) | 3.33 (0.36, 31.26) | 0.27 | **-** | **-** |
| Eczema | 3/17 (18) | 6/29 (21) | 1.17 (0.34, 4.09) | 1.22 (0.26, 5.67) | 0.80 | 0.80 (0.10, 6.56) | 0.83 |
| Food allergy | 0/17 (0) | 1/29 (3) | **-** | **-** | **-** | **-** | **-** |

RR=risk ratio, OR=unadjusted odds ratio, aOR= adjusted odds ratio, API = Asthma predictive index, URTI = upper respiratory tract infection, LRTI = lower respiratory tract infection

^†^Model adjusted for mother’s ethnic group, presence of household smokers, maternal smoking in pregnancy, exclusive breast-feeding to four months, any parental allergic history, any child vitamin supplementation, number of children in the household, age mother left full-time education and baseline concentration of 25(OH)D in maternal blood. n=139 for primary outcome measure

Table S2. Clinical outcomes at age three years in offspring of mothers with baseline 25(OH)D <25nmol/L.

**Daily vitamin D versus control.**

|  | Control | Daily vitamin D | RR | OR | *P* | aOR^†^ | *P* |
| --- | --- | --- | --- | --- | --- | --- | --- |
|  | (%) | (%) | (95% CI) | (95% CI) |  | (95% CI) |  |
| Wheeze ever | 7/24 (29.2) | 4/25 (16) | 0.60 (0.20, 1.77) | 0.46 (0.12, 1.85) | 0.27 | 0.23 (0.04, 1.33) | 0.10 |
| Recurrent wheezing | 4/24 (16.7) | 3/25 (12.0) | 0.72 (0.18, 2.89) | 0.68 (0.14, 3.43) | 0.64 | 0.40 (0.06, 2.76) | 0.35 |
| Wheezing in the last year | 5/24 (20.8) | 3/25 (12.0) | 0.58 (0.15, 2.15) | 0.52 (0.11, 2.46) | 0.40 | - | - |
| Wheeze with positive API | 4/24 (16.7) | 3/25 (12.0) | 0.72 (0.18, 2.89) | 0.68 (0.14, 3.43) | 0.64 | 0.35 (0.04, 2.96) | 0.34 |
| Any bronchodilator use | 2/24 (8.3) | 4/25 (16.0) | 1.92 (0.39, 9.54) | 2.10 (0.35, 12.67) | 0.42 | 2.12 (0.12, 36.28) | 0.60 |
| Eczema ever | 8/24 (33.3) | 7/24 (29.2) | 0.88 (0.38, 2.03) | 0.82 (0.24, 2.80) | 0.76 | 0.16 (0.02, 1.21) | 0.08 |
| Eczema in the last year | 4/24 (16.7) | 5/25 (20.0) | 1.20 (0.37, 3.94) | 1.25 (0.29, 5.35) | 0.76 | 0.59 (0.09, 4.02) | 0.59 |
| Atopy | 4/13 (30.8) | 1/14 (7.1) | 0.23 (0.03, 1.82) | 0.17 (0.02, 1.82) | 0.11 | - | - |
| Allergic rhinitis | 3/24 (12.5) | 3/25 (12.0) | 0.96 (0.21, 4.30) | 0.96 (0.17, 5.27) | 0.96 | 0.68 (0.05, 9.10) | 0.77 |
| Food allergy diagnosis | 2/24 (0.0) | 3/25 (8.0) | 1.44 (0.26, 7.88) | 1.50 (0.23, 9.87) | 0.67 | - | - |
| > 4 URTI/year | 3/24 (12.5) | 6/25 (24.0) | 1.92 (0.54, 6.82) | 2.21 (0.48, 10.09) | 0.30 | 1.03 (0.04, 24.96) | 0.99 |
| LRTI ever | 7/24 (29.2) | 6/24 (25.0) | 0.86 (0.34, 2.18) | 0.81 (0.23, 2.90) | 0.75 | 0.52 (0.10, 2.64) | 0.43 |
| Primary health care record |  |  |  |  |  |  |  |
| Recurrent wheeze | 1/17 (5.9) | 2/16 (12.5) | 2.13 (0.21, 21.24) | 2.29 (0.19, 28.00) | 0.51 | - | **-** |
| Eczema | 3/17 (17.6) | 2/16 (12.5) | 0.71 (0.14, 3.70) | 0.67 (0.10, 4.62) | 0.68 | - | **-** |
| Food allergy | 0/17 (0.0) | 1/16 (6.3) | - | - | - | - | **-** |

RR= Risk ratio, OR=unadjusted odds ratio, aOR= adjusted odds ratio, API = Asthma predictive index, URTI = upper respiratory tract infection, LRTI = lower respiratory tract infection

^†^Model adjusted for mother’s ethnic group, presence of household smokers, maternal smoking in pregnancy, exclusive breast-feeding to four months, any parental allergic history, any child vitamin supplementation, number of children in the household, age mother left full-time education and baseline concentration of 25(OH)D in maternal blood.

Table S3. Clinical outcomes at age three years in offspring of mothers with baseline 25(OH)D <25nmol/L.

Bolus vitamin D versus control.

|  | Control | Bolus vitamin D | RR | OR | *P* | aOR^†^ | *P* |
| --- | --- | --- | --- | --- | --- | --- | --- |
|  | (%) | (%) | (95% CI) | (95% CI) |  | (95% CI) |  |
| Wheeze ever | 7/24 (29.2) | 7/22 (31.8) | 1.09 (0.46, 2.61) | 1.13 (0.32, 3.98) | 0.85 | 1.32 (0.23, 7.63) | 0.76 |
| Recurrent wheezing | 4/24 (16.7) | 5/22 (22.7) | 1.36 (0.42, 4.44) | 1.47 (0.34, 6.37) | 0.61 | 9.95 (0.49, 203.97) | 0.14 |
| Wheezing in the last year | 5/24 (20.8) | 5/22 (22.7) | 1.09 (0.36, 3.27) | 1.12 (0.28, 4.54) | 0.88 | 2.24 (0.29, 17.58) | 0.44 |
| Wheeze with positive API | 4/24 (16.7) | 3/21 (14.3) | 0.86 (0.22, 3.40) | 0.83 (0.16, 4.24) | 0.83 | 2.19 (0.15, 31.74) | 0.57 |
| Any bronchodilator use | 2/24 (8.3) | 5/19 (26.3) | 3.16 (0.69, 14.52) | 3.93 (0.67, 23.10) | 0.11 | 5.52 (0.36, 83.79) | 0.22 |
| Eczema ever | 8/24 (33.3) | 7/19 (36.8) | 1.11 (0.49, 2.50) | 1.17 (0.33, 4.12) | 0.81 | 0.66 (0.11, 3.88) | 0.65 |
| Eczema in the last year | 4/24 (16.7) | 5/19 (26.3) | 1.58 (0.49, 5.08) | 1.79 (0.41, 7.86) | 0.44 | 0.21 (0.001, 32.76) | 0.54 |
| Atopy | 4/13 (30.8) | 3/13 (23.1) | 0.75 (0.21, 2.71) | 0.68 (0.12, 3.87) | 0.66 | - | - |
| Allergic rhinitis | 3/24 (12.5) | 1/19 (5.3) | 0.42 (0.05, 3.73) | 0.39 (0.04, 4.07) | 0.42 | - | - |
| Food allergy diagnosis | 2/24 | 2/19 | 1.26 (0.20, 8.16) | 1.29 (0.17, 10.15) | 0.81 | - | - |
| > 4 URTI/year | 3/24 (12.5) | 6/19 (31.6) | 2.53 (0.72, 8.81) | 3.23 (0.69, 15.20) | 0.13 | 11.76 (0.43, 323.56) | 0.15 |
| LRTI ever | 7/24 (29.2) | 7/19 (36.8) | 1.26 (0.54, 2.98) | 1.42 (0.39, 5.11) | 0.59 | 1.94 (0.36, 10.46) | 0.44 |
| Primary health care record |  |  |  |  |  |  |  |
| Recurrent wheeze | 1/17 (5.9) | 3/13 (23.1) | 3.92 (0.46, 33.52) | 4.80 (0.44, 52.76) | 0.17 | - | - |
| Eczema | 3/17 (17.6) | 4/13 (30.8) | 1.74 (0.47, 6.47) | 2.07 (0.37, 11.53) | 0.40 | - | - |
| Food allergy | 0/17 | 0/13 | - | - | - | - | - |

RR=Risk ratio, aOR= adjusted odds ratio, API = Asthma predictive index, URTI = upper respiratory tract infection, LRTI = lower respiratory tract infection

^†^Model adjusted for mother’s ethnic group, presence of household smokers, maternal smoking in pregnancy, exclusive breast-feeding to four months, any parental allergic history, any child vitamin supplementation, number of children in the household, age mother left full-time education and baseline concentration of 25(OH)D in maternal blood.

Table S4. Sensitivity analysis using imputation for missing primary outcome data.

|  | Control | Treatment group | RR | OR | *P* |
| --- | --- | --- | --- | --- | --- |
|  | (%) | (%) | (95% CI) | (95% CI) |  |
| Scenario assuming no drop-outs wheezed |  |  |  |  |  |
| Control vs Combined | 14/60 (23) | 26/120 (22) | 0.93 (0.52, 1.64) | 0.91 (0.43, 1.90) | 0.80 |
| Control vs Daily | 14/60 (23) | 11/60 (18) | 0.79 (0.39, 1.59) | 0.74 (0.30, 1.79) | 0.50 |
| Control vs Bolus | 14/60 (23) | 15/60 (25) | 1.07 (0.57, 2.02) | 1.10 (0.48, 2.53) | 0.83 |
| Scenario assuming all drop-outs wheezed |  |  |  |  |  |
| Control vs Combined | 24/60 (40) | 38/120 (32) | 0.79 (0.53, 1.19) | 0.70 (0.37, 1.32) | 0.27 |
| Control vs Daily | 24/60 (40) | 15/60 (25) | 0.63 (0.37, 1.07) | 0.50 (0.23, 1.09) | 0.08 |
| Control vs Bolus | 24/60 (40) | 23/60 (38) | 0.96 (0.61, 1.50) | 0.93 (0.45, 1.94) | 0.85 |

RR=Risk ratio, OR=Odds ratio, CI= Confidence intervals

Table S5. Cord vitamin D levels and clinical outcomes at age 3 years

| Outcome | n | Outcome absent | n | Outcome present | Unadjusted Mean | p | Adjusted^1^ mean | p |
| --- | --- | --- | --- | --- | --- | --- | --- | --- |
|  |  | Mean ± SD |  | Mean ± SD | difference |  | difference |  |
|  |  |  |  |  | (95% CI) |  | (95% CI) |  |
| Wheeze | 88 | 3.20 ± 0.52 | 34 | 3.07 ± 0.43 | -0.13 (-0.33, 0.07) | 0.19 | -0.07 (-0.26, 0.11) | 0.43 |
| Atopy | 59 | 3.19 ± 0.49 | 14 | 3.28 ± 0.44 | 0.08 (-0.20, 0.37) | 0.56 | 0.10 (-0.17, 0.37) | 0.47 |
| Eczema | 82 | 3.14 ± 0.51 | 34 | 3.17 ± 0.48 | 0.03 (-0.23, 0.17) | 0.74 | 0.05 (-0.13, 0.23) | 0.56 |
| Any LRTI | 84 | 3.17 ± 0.52 | 31 | 3.16 ± 0.45 | -0.007 (-0.20, 0.22) | 0.95 | -0.10 (-0.29, 0.09) | 0.30 |
| > 4 URTI/year | 95 | 3.19 ± 0.52 | 22 | 3.03 ± 0.41 | -0.16 (-0.40, 0.07) | 0.17 | -0.11 (-0.32, 0.11) | 0.33 |

LRTI (Lower respiratory tract infection), URTI (Upper respiratory tract infection).

^1^Adjusted for treatment group, mother’s ethnic group, presence of household smokers, maternal smoking in pregnancy, exclusive breast-feeding to four months, any parental allergic history, any child vitamin supplementation, number of children in the household, and age mother left full-time education.

**Table S6. Lung function at age three years. Combined vitamin D groups versus control.**

| Outcome | n | Control | n | Combined | Unadjusted Mean | *P* | Adjusted Mean | *P* |
| --- | --- | --- | --- | --- | --- | --- | --- | --- |
|  |  | Mean ± SD |  | Vitamin D | difference |  | difference^†^ |  |
|  |  |  |  | Mean ± SD | (95% CI) |  | (95% CI) |  |
| R10 (kPa/(L/s)) | 13 | 0.97 ± 0.14 | 38 | 0.93 ± 0.16 | -0.04 (-0.14, 0.06) | 0.44 | -0.05 (-0.17, 0.06) | 0.38 |
| R20 (kPa/(L/s)) | 13 | 0.82 ± 0.12 | 38 | 0.77 ± 0.13 | -0.05 (-0.13, 0.04) | 0.26 | -0.05 (-0.14, 0.05) | 0.31 |
| F_res_ (Hz) | 13 | 25.0 ± 2.17 | 38 | 24.8 ± 3.28 | -0.28 (-2.25, 1.68) | 0.77 | -0.49 (-2.66, 1.68) | 0.65 |
| AX (kPa/L) | 13 | 3.89 ± 1.59 | 38 | 3.90 ± 1.69 | 0.02 (-1.06, 1.10) | 0.97 | -0.19 (-1.35, 0.97) | 0.74 |
| R10% (%) | 10 | -10.66 ± 8.12 | 29 | -9.74 ± 10.50 | -0.91 (-8.33, 6.50) | 0.80 | -0.55 (-8.50, 7.38) | 0.89 |
| R20% (%) | 10 | -6.37 ± 10.52 | 29 | -5.89 ± 9.31 | -0.48 (-7.62, 6.67) | 0.89 | -1.36 (-8.49, 5.78) | 0.70 |
| F_res_% (%) | 10 | -12.07 ± 8.59 | 29 | -10.92 ± 11.22 | -1.15 (-9.05, 6.76) | 0.77 | 0.96 (-8.70, 10.61) | 0.84 |
| AX% (%) | 10 | -34.20 ± 23.30 | 29 | -31.94 ± 26.92 | -2.26 (-21.64, 17.12) | 0.81 | 3.80 (-21.39, 28.99) | 0.76 |

R10 and R20 = resistance at 10 and 20 Hz, F_res_ = resonant frequency, AX = area under the reactance curve. Variables labelled ‘post’ refer to repeat lung function assessment taken 15 minutes after 400 mcg inhaled salbutamol. All lung function data were acquired using impulse oscillometry.

^†^Model adjusted for mother’s ethnic group, presence of household smokers, maternal smoking in pregnancy, exclusive breast-feeding to four months, any parental allergic history, any child vitamin supplementation, number of children in the household, age mother left full-time education, baseline concentration of 25(OH)D in maternal blood

**Table S7. Measures of allergic inflammation at age three years. Combined vitamin D groups versus control.**

| Outcome | n | Control | n | Combined vitamin D | Unadjusted mean | *P* | Adjusted mean | *P* |
| --- | --- | --- | --- | --- | --- | --- | --- | --- |
|  |  | Mean ± SD |  | Mean ± SD | difference |  | difference^†^ |  |
|  |  |  |  |  | (95% CI) |  | (95% CI) |  |
| Ln IgE | 27 | 3.74 ± 1.29 | 59 | 3.44 ± 1.52 | -0.30 (-0.97, 0.37) | 0.38 | -0.39 (-1.15, 0.36) | 0.30 |
| IgE^‡^ (kU/L) | 27 | 42.1 ± 3.62 | 59 | 31.20 ± 4.57 | 0.74 (0.38, 1.45) | 0.38 | 0.68 (0.32, 1.43) | 0.30 |
| eNO (ppb) | 20 | 23.50 ± 12.71 | 42 | 19.30 ± 9.28 | -4.18 (-9.88, 1.52) | 0.15 | -3.34 (-9.43, 2.75) | 0.28 |
| Ln Eos | 27 | 1.11 ± 0.70 | 53 | 1.01 ± 0.69 | -0.10 (-0.43, 0.22) | 0.53 | -0.25 (-0.61, 0.11) | 0.18 |
| Eos^§^ (%) | 27 | 3.03 ± 2.01 | 53 | 2.75 ± 1.99 | 0.90 (0.65, 1.25) | 0.53 | 0.78 (0.54, 1.12) | 0.18 |

eNO = exhaled nitric oxide, Eos = eosinophil count as a % of total white cell count

^†^Model adjusted for mother’s ethnic group, presence of household smokers, maternal smoking in pregnancy, exclusive breast-feeding to four months, any parental allergic history, any child vitamin supplementation, number of children in the household, age mother left full-time education, baseline concentration of 25(OH)D in maternal blood.

^‡^Ln IgE result transformed into geometric mean ± SD and multiplicative mean difference with 95% CI

^§^Ln Eos result transformed into geometric mean ± SD and multiplicative mean difference with 95% CI

**REFERENCES**

1. Beydon N, Davis SD, Lombardi E, Allen JL, Arets HG, et al. (2007) An official American Thoracic Society/European Respiratory Society statement: pulmonary function testing in preschool children. Am J Respir Crit Care Med 175: 1304-1345.

2. (2005) ATS/ERS recommendations for standardized procedures for the online and offline measurement of exhaled lower respiratory nitric oxide and nasal nitric oxide, 2005. Am J Respir Crit Care Med 171: 912-930.

3. Baraldi E, de Jongste JCi (2002) Measurement of exhaled nitric oxide in children, 2001. The European respiratory journal 20: 223-237.
